# Impacts of High Intra- and Inter-Individual Variability in Tacrolimus Pharmacokinetics and Fast Tacrolimus Metabolism on Outcomes of Solid Organ Transplant Recipients

**DOI:** 10.3390/jcm9072193

**Published:** 2020-07-11

**Authors:** Charat Thongprayoon, Panupong Hansrivijit, Karthik Kovvuru, Swetha R. Kanduri, Tarun Bathini, Aleksandra Pivovarova, Justin R. Smith, Wisit Cheungpasitporn

**Affiliations:** 1 Division of Nephrology, Department of Medicine, Mayo Clinic, Rochester, MN 55905, USA; charat.thongprayoon@gmail.com; 2 Department of Internal Medicine, University of Pittsburgh Medical Center Pinnacle, Harrisburg, PA 17105, USA; hansrivijitp@upmc.edu; 3 Division of Nephrology, Department of Medicine, University of Mississippi Medical Center, Jackson, MS 39216, USA; kkovvuru@umc.edu (K.K.); skanduri@umc.edu (S.R.K.); 4 Department of Internal Medicine, University of Arizona, Tucson, AZ 85724, USA; tarunjacobb@gmail.com; 5 Department of Medicine, University of Mississippi Medical Center, Jackson, MS 39216, USA; apivovarova@umc.edu (A.P.); jsmith20@umc.edu (J.R.S.)

**Keywords:** tacrolimus, calcineurin inhibitors, FK506, transplantation, kidney transplantation, immunosuppression, pharmacokinetic, C/D ratio, fast tacrolimus metabolizers

## Abstract

Tacrolimus is a first-line calcineurin inhibitor (CNI) and an integral part of the immunosuppressive strategy in solid organ transplantation. Being a dose-critical drug, tacrolimus has a narrow therapeutic index that necessitates periodic monitoring to maintain the drug’s efficacy and reduce the consequences of overexposure. Tacrolimus is characterized by substantial intra- and inter-individual pharmacokinetic variability. At steady state, the tacrolimus blood concentration to daily dose ratio (C/D ratio) has been described as a surrogate for the estimation of the individual metabolism rate, where a low C/D ratio reflects a higher rate of metabolism. Fast tacrolimus metabolism (low C/D ratio) is associated with the risk of poor outcomes after transplantation, including reduced allograft function and survival, higher allograft rejection, CNI nephrotoxicity, a faster decline in kidney function, reduced death-censored graft survival (DCGS), post-transplant lymphoproliferative disorders, dyslipidemia, hypertension, and cardiovascular events. In this article, we discuss the potential role of the C/D ratio in a noninvasive monitoring strategy for identifying patients at risk for potential adverse events post-transplant.

## 1. Introduction

Calcineurin inhibitor (CNI)-based immunosuppression is a regularly used therapeutic regimen in solid organ transplantation (SOT), tacrolimus (also known as FK506) being the front-running CNI [1,2] and mainstay of triple immunosuppressive drug regimens [3]. Before extended-release formulations became available, tacrolimus was traditionally given twice daily [4,5,6,7,8,9,10,11,12,13] (Table 1). Immediate-release formulations have highly variable absorption profiles and absolute bioavailability, ranging from 5 to 93%, with the average being 25 to 30% [14,15,16,17]. The tacrolimus protein binding rate is approximately 99%, and it also extensively binds to red blood cells. Protein binding is noted to be primarily with α1-acid glycoprotein and albumin [16,18]. Tacrolimus undergoes extensive hepatic metabolism, and less than 1% of the unaltered parent drug is eliminated [3].

The narrow therapeutic index of tacrolimus necessitates the frequent monitoring of the whole-blood concentration to achieve optimal therapeutic levels while drug toxicity [17,23,24,25,26]. Even a small variance in the dose or concentration can lead to therapeutic failure. Nonetheless, the therapeutic advantages of tacrolimus outweigh its major disadvantages such as the large inter-patient pharmacokinetic (PK) variability and potential risk of drug interactions with co-administrated medications [27]. While supra-therapeutic levels of tacrolimus can lead to neurotoxicity, nephrotoxicity, hypertension, and post-transplant diabetes, sub-therapeutic levels have been associated with allograft rejection [28].

Tacrolimus is primarily metabolized in the liver and small intestine through cytochrome P450 isoforms 3A4 and 3A5 (CYP3A4/5) [29]. Furthermore, tacrolimus is a substrate for P-glycoprotein (P-gp), a membrane efflux pump that actively transports the drug out of cells, which also contributes significantly to PK variability [30]. Inter-individual PK variability can be affected by the amount of time passed since the transplant, patient demographics (age and race), hepatic and renal function, the hematocrit level, food administration, concomitant medications (corticosteroids, antifungals, calcium channel blockers, etc.), and the genotype for metabolic enzymes [16,31] (Table 2). For instance, the CYP3A5*3 variant minor allele frequency (MAF) is estimated to be as high as 95% among Caucasians, while the African American population carries it at a rate of up to 33% [32]. A few studies have reported that mycophenolate mofetil co-administration may influence the metabolism of tacrolimus by possibly competing for CYP3A [33,34]. Relatedly, corticosteroids induce CYP3A4 and P-gp pathways that may potentially influence tacrolimus metabolism, yet the data are conflicting [35]. Increased tacrolimus levels upon the de-escalation of the dose or withdrawal of steroids have been reported [36,37]. In addition, by influencing the conversion of uridine diphosphoglucuronosyltransferase to the glucuronide metabolite of mycophenolicacid (MPA), tacrolimus may affect mycophenolicacid (MPA) levels [38].

## 2. Intra-Individual and Inter-Individual Tacrolimus PK Variability

Therapeutic drug monitoring (TDM) is essential for achieving therapeutic trough concentrations. Despite the fact that monitoring blood trough concentrations is an effective method for adjusting tacrolimus oral doses, clinical studies have reported that it may not be valuable for future dosage estimation and may also not be an accurate assessment of overall drug exposure among various individuals [17,23,24,25,26]. It is sometimes challenging to achieve and maintain target tacrolimus trough concentrations despite periodic tacrolimus TDM [3]. Hence, the determination of the area under the curve (AUC) over a dosing interval is generally considered the best indicator of optimal dosing, though multiple blood samples may be required, which, for practical and financial reasons, limits its clinical utility [38].

As discussed earlier, tacrolimus has a narrow therapeutic index and substantial inter-individual PK variability with standard weight-based dosing [56,57]. Moreover, tacrolimus also has a high intra-individual variability (IPV), resulting in sub-therapeutic and supra-therapeutic concentrations despite comparable dosing [25]. The coefficient of variation (CV) serves as a well-established biomarker of adherence in pediatric and adult groups of kidney transplant recipients [58]. It is derived by dividing the standard deviation (SD) of a number of serial pre-dose concentrations by the mean of these tacrolimus measurements. The CV is also utilized to assess IPV [18]. The IPV in the trough blood levels of tacrolimus represented by the CV is also a notable prognostic factor for graft function attributable to T-cell- and humoral-mediated rejection as well as vascular changes [59,60,61,62]. Kaya Aksoy et al., for a cohort of 67 pediatric kidney transplant recipients, reported that a tacrolimus CV cutoff value of 32% is considerably precise in identifying the further development of de novo donor-specific antibody (dnDSA) (AUC 0.713) [63]. During a follow-up period, a tacrolimus CV over 32% is associated with a higher percentage of development of dnDSA; 67% vs. 31% during 6 to 12 months, and 83% vs. 47% after 1 year of transplantation. Shuker et al. described that a high tacrolimus IPV in conjunction with lower tacrolimus pre-dose concentrations at 1 year post-transplantation correlates with adverse outcomes, including allograft rejection, the doubling of serum creatinine, and allograft loss [64]. Similarly, in a study by Rozen-Zvi et al., patients with a combination of low drug level exposure and high tacrolimus time-weighed variability had lower graft survival rates than patients with other exposure and variability combinations [65].

To date, multiple studies have confirmed that in kidney transplant recipients, sub-therapeutic tacrolimus levels or high tacrolimus IPV can result in increased dnDSA formation [25,65,66,67,68,69,70,71]. In the modern era, there has been increasing interest in the identification and validation of genetic variations that contribute to IPV [72,73]. Germline mutations in ATP-binding cassette B1 gene (ABCB1) and CYP3A4/5 probably contribute to interindividual tacrolimus PK variability [3,17,74]. Single nucleotide polymorphisms (SNPs) in CYP3A5 could contribute 40%–50% of inter-individual PK variability [75,76]. Two intragenic CYP3A4 SNPs are hypothesized to cause inter-individual PK variability [72,73]. Genetic variants in drug transporters may also add to tacrolimus’ PK variability. The ABCB1 gene, immensely expressed on hepatocytes and enterocytes, encodes P-gp; ABCB1 SNPs potentially add to inter-individual tacrolimus absorption and nephrotoxicity, respectively [3]. Renal tubular epithelial cells express P-gp, and SNPs have been associated with a variable risk of tacrolimus-induced nephrotoxicity [39,77]. Although genetic variability and environmental factors affect tacrolimus’ IPV, non-adherence is still the prevailing cause of high IPV [18,78,79,80].

Graft function and survival have been associated with tacrolimus trough levels and their variability [57,67,81,82,83]. Eminent studies by Sapir-Pichhadze et al. showed that a higher standard deviation in tacrolimus levels correlates with an increased likelihood of unfavorable endpoints, including allograft rejection, transplant glomerulopathy, and allograft loss [78]. Similar results were reproduced by several other studies [64,79,80,81]. Sablik et al. reported that even though high tacrolimus IPV was not associated with the incidence of chronic active antibody-mediated rejection (ABMR), high tacrolimus IPV, as compared to low IPV, showed a significant association with lower allograft survival in recipients with chronic active ABMR [84].

## 3. Fast Tacrolimus Metabolizers at Risk

Given its predictive value regarding post-transplant outcomes, there is an increasing interest in analyzing the rate of tacrolimus metabolism [83,85,86,87,88,89,90]. At steady state, the tacrolimus concentration to daily dose ratio (C/D ratio) has been described as a surrogate for the individual rate of tacrolimus metabolism, where a low C/D ratio is indicative of a higher rate of tacrolimus metabolism [88,90,91,92]. The C/D ratio is calculated using the following formula [62,88,93]:

C/D ratio (ng/mL × 1/mg) = blood tacrolimus trough concentration (ng/mL)/daily tacrolimus dose (mg)

Since tacrolimus trough levels and their corresponding doses are routinely recorded, calculating the C/D ratio could be utilized as a valuable tool for tacrolimus metabolism rate estimation. CYP3A5 expressers need higher doses of tacrolimus to reach comparable trough levels, even though CYP3A5 expression alone might not necessarily reflect fast metabolism of tacrolimus [94]. The tacrolimus metabolization phenotype has a fundamental effect on graft survival, which cannot be totally explained by this particular CYP3A5*1 genotype [56,83,95,96]. The tacrolimus C/D ratio might assist in identifying patients who are more susceptible to rapid alterations in tacrolimus blood levels. However, the C/D ratio is only a minor reflection of medication non-adherence, unlike tacrolimus IPV and the time in therapeutic range [25].

The C/D ratio has particularly been studied as a potential prognostic factor for renal allograft function after solid organ transplantation. Allograft function and allograft rejection have been reported to correlate with the C/D ratio [62,85,88]. Fast tacrolimus metabolism (a C/D ratio <1.05 ng/mL/mg) is correlated with inferior outcomes after kidney transplantation secondary to a higher rejection rate and risk of CNI nephrotoxicity (CNIT) [62,88,89] (Table 3). A low immediate-release tacrolimus C/D ratio is linked with higher C2 tacrolimus blood concentrations (2 h after tacrolimus intake) despite comparable trough levels in recipients with high C/D ratios [89]. In kidney and liver transplant recipients, a low C/D ratio strongly correlated with an increased risk of CNIT along with rapid kidney function decline [62,85,87,89,90]. In addition, the C/D ratio is a superior and early predictor of death-censored graft survival (DCGS). In a multivariate analysis, a C/D ratio below 1.05 was associated with death-censored graft loss risk elevation by a factor of 2.26. Tacrolimus clearance fluctuates more in the early post-transplant period owing to changes in gastrointestinal mobility, albumin and hematocrit levels, and steroid dosing [97]. Therefore, it is possible to identify individuals at risk one to three months following transplantation [83]. Both the time-dependent C/D ratio and early C/D ratios play similar roles in predicting the risk of graft loss. Notably, even a single C/D ratio calculated in stable patients three months after kidney transplant predicted outcomes, as it is relatively stable during those postoperative months [62,83]. Recently, Jouve et al. reported that the time-dependent and early tacrolimus C/D ratios appear to be independent predictors of DCGS [83].

Interestingly, while the tacrolimus trough blood concentration was only associated with the onset of de novo hypertension and cardiovascular events, fast-metabolizers are prone to developing more de novo dyslipidemia and insulin requiring diabetes along with de novo hypertension and cardiovascular events [62]. Furthermore, a very recent study showed that fast-metabolizers have a higher risk of developing post-transplant lymphoproliferative disorders (PTLD), although this association calls for further studies to validate the results [98]. The identification of fast metabolizers and optimization of tacrolimus formulations may contribute to beneficial therapeutic strategies in efforts to improve graft survival. With that being said, we highly encourage further studies to investigate the role of the C/D ratio and its association with cardiovascular risks and PTLD [83]. 

Lastly, exploring the concentrations of tacrolimus metabolites may also be of clinical value in the interpretation of the C/D ratio’s significance: individuals with a high C/D ratio might utilize different metabolic pathways, resulting in variable concentrations of tacrolimus metabolites, yielding divergent safety profiles. Furthermore, the C/D ratio is a surrogate of tacrolimus metabolite concentrations [99,100,101,102]. As reported by the ASERTAA (A Study of Extended Release Tacrolimus in African Americans) study, the C/D ratio varies depending on the tacrolimus formulation (extended-release tacrolimus (ENVARSUS XR^®^) versus immediate-release tacrolimus (PROGRAF^®^)) (Table 1) [103]. Using extended-release formulations of the drug will expectedly increase the C/D ratio due to a decreased total daily dose; however, whether this would impact graft survival is still under investigation. Additionally, future studies implementing CNI-free immunosuppression or minimizing tacrolimus and impacts on graft outcomes need to be evaluated among fast metabolizers [104].

## 4. Conclusions

In summary, the C/D ratio is a simple and valuable clinical tool in identifying patients who might benefit from immunosuppressive regimen adjustment. When patients require higher daily doses of tacrolimus to achieve therapeutic trough levels (i.e., a C/D ratio < 1), they may have a higher risk of subsequent graft loss. A combination of various tacrolimus monitoring strategies including determining the C/D ratio, IPV, and mean pre-dose concentration may identify solid organ transplant recipients at risk for poor outcomes. 

## Figures and Tables

**Table 1:** Pharmacokinetics of available formulations of tacrolimus in the United States.

Trade Name	Active Ingredient	Oral Dose *	Pharmacokinetic Parameters			Half-Life (h) †,§	Metabolism	References
			Cmax (ng/mL) †	Tmax (h) ‡	AUC_24_ (ng h/mL) †			
Astagraf XL	Extended-release tacrolimus; once daily	0.20 mg/kg	26.0 ± 13.7	3.0 (2–24)	372 ± 202	31.9 ± 10.5	CYP3A4, 3A5	[19]
Envarsus XR	Extended-release tacrolimus; once daily	0.14 mg/kg	11.8 ± 7.2	8.0 (4–24)	138 ± 80	31.9 ± 10.5	CYP3A4, 3A5	[20]
Hecoria	Tacrolimus; twice daily	0.20 mg/kg	19.2 ± 10.3	3.0 (N/A)	203 ± 42	31.9 ± 10.5	CYP3A4, 3A5	[21]
Prograf	Tacrolimus; twice daily	0.20 mg/kg	19.2 ± 10.3	3.0 (N/A)	203 ± 42	31.9 ± 10.5	CYP3A4, 3A5	[22]

Cmax, maximum concentration; Tmax, time-to-peak concentration; AUC_24_, 24-hour area under curve; N/A, not available. * data obtained from kidney transplant patients. † mean, ± standard deviation. ‡ median (interquartile range). § represents elimination half-life measured by radioactivity.

**Table 2:** Factors associated with alteration of tacrolimus trough levels.

Factor(s) Reducing Tacrolimus Trough Level				Factor(s) Increasing Tacrolimus Trough Level			
Factor(s)	Example	Description	References	Factor(s)	Example	Description	References
CYP3A4*1B allele	-	Results in the hyperactivity of CYP3A4, involved in tacrolimus metabolism	[39,40]	CYP3A5*3 allele	Native Americans	Results in hypoactivity of CYP3A5, involved in tacrolimus metabolism	[41,42,43,44]
CYP3A5*3, CYP3A5*6, CYP3A5*7 variants	African Americans	Results in the hyperactivity of CYP3A5, involved in tacrolimus metabolism	[45]	CYP3A4 inhibitors	Ketoconazole (>90% inhibition); Cyclosporin A, nifedipine (>40% inhibition); Diltiazem, erythromycin, fluconazole, rifampicin (>10% inhibition)		[46]
ABCB1 genotype	Chinese	Encodes for p-glycoprotein, a protein responsible for the intestinal excretion of tacrolimus	[47,48,49]	Diarrhea	Case reports	Intestinal epithelial cells may be destroyed, abrogating excretion via P-glycoproteins	[50,51,52]
High fat meals	-	Reduces tacrolimus absorption	[53]	Biliary obstruction	Case reports	99% of tacrolimus is excreted via bile. Liver dysfunction or bile secretion defects could result in tacrolimus toxicity	[54]
There are insufficient data to determine whether celiac disease, gastroparesis, or inflammatory bowel disease would alter tacrolimus bioavailability				Hepatic dysfunction	Cirrhosis, hepatic veno-occlusive disease		[55]

**Table 3:** Poor outcomes associated with fast tacrolimus metabolism (low C/D ratio).

Reported Poor Outcomes Associated with Fast Tacrolimus Metabolism (Low C/D Ratio)
Reduced allograft graft function Allograft rejection CNI nephrotoxicity Faster decline in kidney function Reduced death-censored graft survival Dyslipidemia Hypertension Cardiovascular events Post-transplant lymphoproliferative disorders
